# Paleohistology and Lifestyle Inferences of a Dyrosaurid (Archosauria: Crocodylomorpha) from Paraíba Basin (Northeastern Brazil)

**DOI:** 10.1371/journal.pone.0102189

**Published:** 2014-07-17

**Authors:** Rafael César Lima Pedroso de Andrade, Juliana Manso Sayão

**Affiliations:** 1 Graduate Student/Programa de Pós-Graduação (CTG), Universidade Federal de Pernambuco, Recife, Brazil; 2 Centro Acadêmico de Vitória, Universidade Federal de Pernambuco, Bela Vista, Vitória de Santo Antão, Pernambuco, Brazil; University of Zurich, Switzerland

## Abstract

Among the few vertebrates that survived the mass extinction event documented at the Cretaceous–Paleocene boundary are dyrosaurid crocodylomorphs. Surprisingly, there is little information regarding the bone histology of dyrosaurids, despite their relatively common occurrence in the fossil record, and the potential to gain insight about their biology and lifestyle. We provide the first description of the long bone histology of the dyrosaurids. Specimens were collected from the Maria Farinha Formation, in the Paraíba Basin of northeast Brazil. Thin sections of a right femur and left tibia were made. In the left tibia, the cortex consists of lamellar-zonal bone with five lines of arrested growth (LAGs), spaced ∼300 µm apart. The tibia contains a small to medium-sized organized vascular network of both simple vascular canals and primary osteons that decrease in density periostially. The femur exhibits a similar histological pattern overall but has double-LAGs, and an EFS layer (the latter is rare in living crocodylians). Secondary osteons occur in the deep cortex near and inside the spongiosa as a result of remodeling in both bones. This tissue pattern is fairly common among slow-growing animals. These specimens were a sub-adult and a senescent. Patterns in the distribution of bone consistent with osteosclerosis suggest that these animals probably hada fast-swimming ecology. Although these results are consistent with the histology in anatomically convergent taxa, it will be necessary to make additional sections from the mid-diaphysis in order to assign their ecology.

## Introduction

Among the few vertebrates that survived the mass extinction event documented at the Cretaceous–Palaeogene boundary are the dyrosaurids [1], which represent an extinct lineage of Neosuchia. Dyrosaurids are found in transitional marine sediments from the Late Cretaceous to Lower Eocene [2], and they exhibit the primary feature of a long snout [3], [4]. The family was named by Giuseppe de Stefano in 1903 [5] for the genus *Dyrosaurus*, referring to the locality where the holotype was found, Djebel Dyr, Algeria.

**Figure 1:**
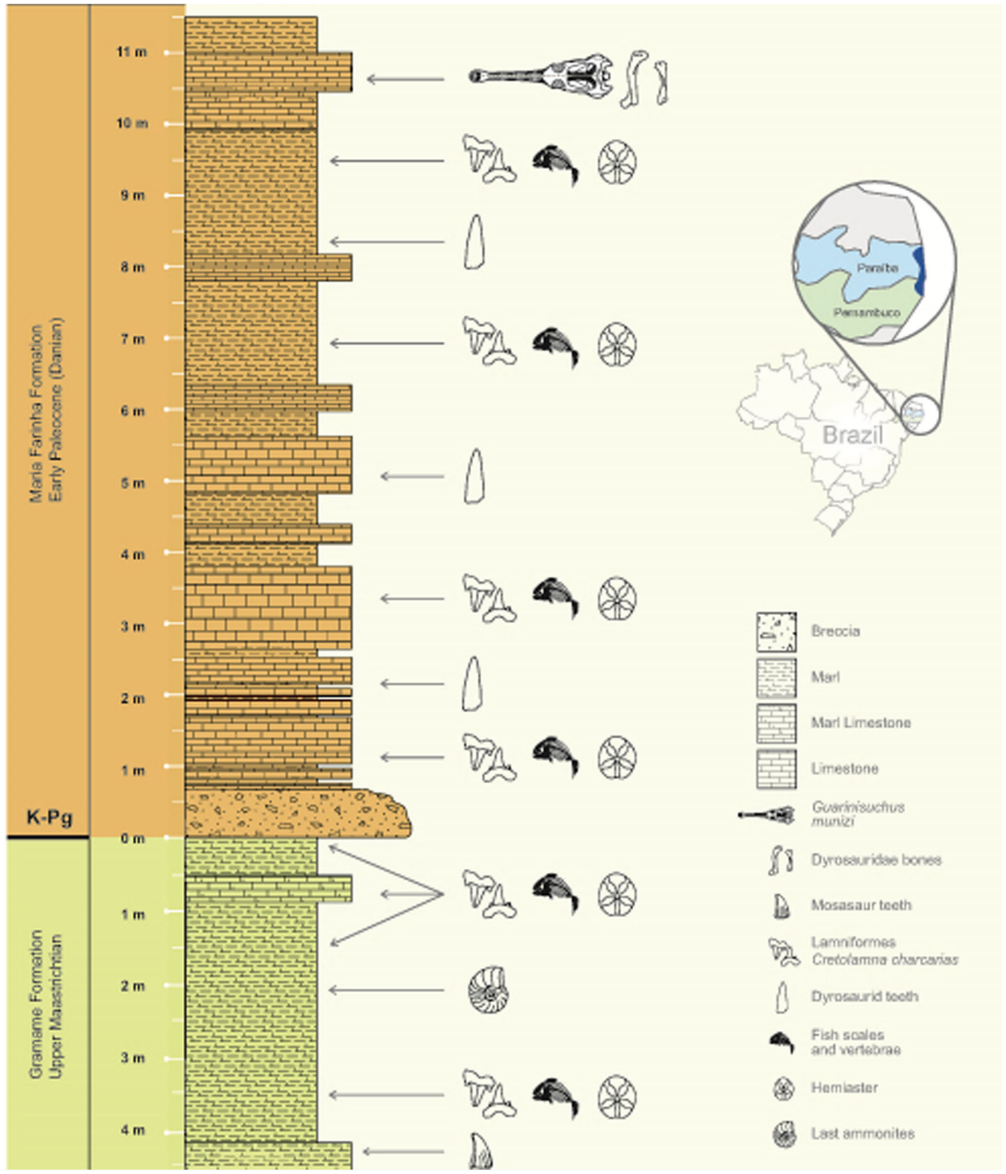
Location of the Paraíba Basin and stratigraphic section of the Maria Farinha Formation at the Poty Quarry, located in the state of Pernambuco, close to the city of Recife, in northeast Brazil (Redrawn from Barbosa et al. 2008 [11]). This section indicates each level of composition (marls, marly-limestones and limestones) and their fossil contents. The position where the dyrosaurid bones were found is indicated above.

In South America, dyrosaurids were previously known only from fragmentary material [6], [7], [8], [9], several cranial elements briefly mentioned in the literature [10], and by the description of an almost complete skull and part of the jaw, ulna, cervical and caudal vertebrae, ribs, dermal scutes and isolated teeth belonging to *Guarinisuchus munizi* [11]. The holotype of *G. munizi* was collected from the Maria Farinha Formation (Paleocene) in the Poty Quarry, which is located close to Recife in northeastern Brazil (Figure 1) [11]. The bones at this site exhibit excellent external preservation in three dimensions, and preserve the internal microstructures as well.

Examination of the microstructure of bones enhance the simple morphological description of the specimens; this approach is a method to obtain certain types of information about the biology of extinct animals, for example, the presence of adaptations to a lifestyle, growth rates, and indications of its ontogenetic stage [12], [13], [14]. The bone structure is composed of mineralized connective tissue (bone tissue) that was produced by hydroxyapatite deposition [15], crystalline calcium phosphate and, in the inner parts, by osteocytes and numerous channels in the form of blood and lymphatic vessels. After death, the organic components, including the cells and blood vessels, decomposed, while the inorganic portion became fossilized, thereby maintaining the bones microstructures and preserving the shape of the decomposed components [16].

Additionally, histological examination has established structural modifications of bone in tetrapod taxa secondarily adapted to life in water, including both fossil and recent taxa [17]. Within this large available data set, the only published thin sections of dyrosaurid bones are of the vertebrae and skull of *Dyrosaurus phosphaticus* [18]. Thus, the paleohistology of dyrosaurids remains largely unexplored. In this study, we present the first histological description of dyrosaurid limb bones, belonging to specimens from the Poty Quarry, Maria Farinha Formation.

## Materials and Methods

### Ethics Statement

No permits were required for the described study, which complied with all relevant regulations. The author JMS collected these materials with Dr. Antônio Barbosa. No permission is required to use them in research since JMS is the curator of the collection were the material is housed.

### Specimens

Two specimens were selected for sampling: the proximal third of a right femur (CAV 0010-V) and the distal portion of a left tibia including the metaphysis (CAV 0011-V). Both specimens are from the paleontological collection of the Centro Acadêmico de Vitória (Universidade Federal de Pernambuco), Vitória de Santo Antão, Pernambuco, Brazil.

The femur and tibia sampled here were found at the type locality of *Guarinisuchus munizi.* They were collected a few months after the holotype, from the same horizon but in rolled blocks. It was not possible to identify whether or not they belong to the same individual, so each element was considered to belong to distinct specimens. They were found associated with an elongated mandible clearly belonging to a dyrosaurid and also near to other isolated appendicular elements. This strongly suggests that the bones sampled here also belong to a dyrosaurid.

The overall crocodyliform record from South American Paleocene deposits is rather slim in both the number of specimens and taxa recovered. Only three crocodyliform lineages cross the K-Pg boundary in South America: the marine Dyrosauridae, and two true crocodylians, the semiaquatic alligatoroids [19]–[21] and the terrestrial sebecosuchians (e.g. [22]). In the Paraíba Basin, the only crocodyliforms that have been recovered are from the family Dyrosauridae [23]. In fact, neither sebecosuchians nor alligatoroids have been reported from all of northeastern Brazil. The Sebecosuchia have a Gondwanan distribution, but in Brazil they are almost entirely restricted to the Mesozoic [24], with one reported occurrence in the Paleocene deposits of São José de Itaboraí Basin [25]. The lack of other crocodyliforms in the age or proximity of Maria Farinha Formation supports their taxonomic assignment to Dyrosauridae. It cannot be confirmed whether or not they belong to *G. munizi*, for which femoral or tibial autapomorphies are not known, but this is a strong possibility as they were collected from the type locality.

### Geological Setting

The Paraíba basin, previously known as the Paraíba-Pernambuco Basin, occupies an exposed area of approximately 7600 km^2^ and a submerged area of approximately 31,400 km^2^, extending on the continental shelf down to the bathymetric quota of 3000 m [29].

In this basin, the K-Pg boundary represents the contact between the Gramame (Maastrichtian) and Maria Farinha (Danian) Formations (Fig. 1). The Maria Farinha Formation is composed of limestones, marly limestones and thick levels of marls in its lower portion, while dolomitic limestones containing fossil reefs and lagunal reefs characterize its upper portion [30], [31]. This formation exhibits the regressive characteristics of high- to low-energy oscillations.

### Slide Preparation

For this analysis, the distal metaphysis of the left tibia and the proximal portion of the right femoral diaphysis were sampled. Typically, the mid-diaphysis is sampled in paleohistological studies, as this region preserves more cortical bone and more of the growth record [26]. Unfortunately, the mid-diaphyses were not preserved in either bone, so the elements were sampled as close as possible to the midshaft.

**Figure 2:**
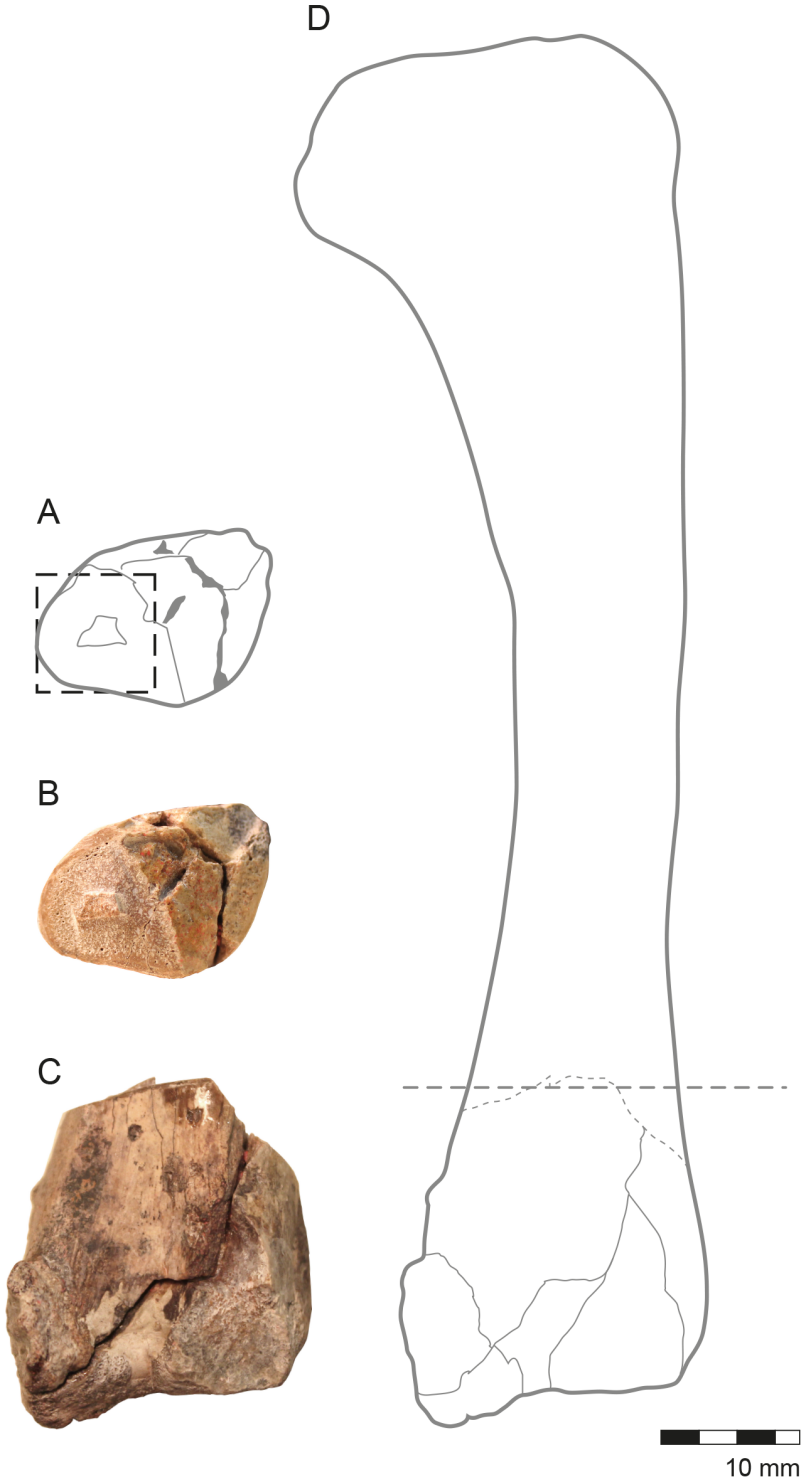
Aspects of external and internal anatomy in the left tibia, CAV 0011-V. (A) The dotted square represents the region where the analysis and images have been made. (B) A view of the section made for sample collection, which corresponds to a distal metaphiso-epiphyseal portion where the cortex is thinner than in the diaphyseal portion. (C) Posterior view of the distal portion of the tibia, all that was preserved in this element. (D) Reconstruction of the complete tibia; the dotted line indicates where the cut was made for the sample collection.

To prepare the histological slides, a 0.5 cm sample was obtained from each specimen (femur and tibia). The diaphysis of the femur was sampled just distal to the fourth trochanter, and the tibia was sampled from the distal metaphysis (Figure 2). Prior to sampling, both bones had been mechanically prepared with the use of airscribes and manual tools. No external surface damage resulting from preparation was observed in neither of the specimens. To preserve the external morphological information of the specimens, molds in silicon rubber (RTV CAL/N - ULTRALUB QUÍMICA LTDA, São Paulo, Brazil) and resin casts (RESAPOL T-208 catalyzed with BUTANOX M50 - IBEX QUIMICOS E COMPÓSITOS, Recife, Brazil) were produced. The bones were subsequently measured and photographed according to the protocol proposed by Lamm (2013) [27].

Thin sections were produced using standard fossil histology techniques [27], [28]. The samples were embedded in epoxy clear resin RESAPOL T-208 catalysed with BUTANOX M50 and cut with a diamond-tipped blade on a saw (multiple brands). Next, the mounting-side of the sections were wet-ground using a metallographic polishing machine (AROPOL-E, Arotec LTDA) with Arotec abrasive papers of increasing grit size (60/P60, 120/P120, 320/P400, 1200/P2500) until a final thickness of ∼30–60 microns was reached.

### Imaging and Image Analysis

To observe the histological structures, an optical microscope in transmitted light mode with parallel/crossed nicols and fluorescence filters were used to enhance birefringence.

Representative histological images were taken using an AxioCam digital sight camera (Zeiss Inc., Barcelona, Spain) mounted to an Axio Imager.M2 transmitted light microscope (Zeiss Inc. Barcelona, Spain). Images were taken at 5× and 10× total magnification.

## Results

### Histological features

#### Tibia

**Figure 3:**
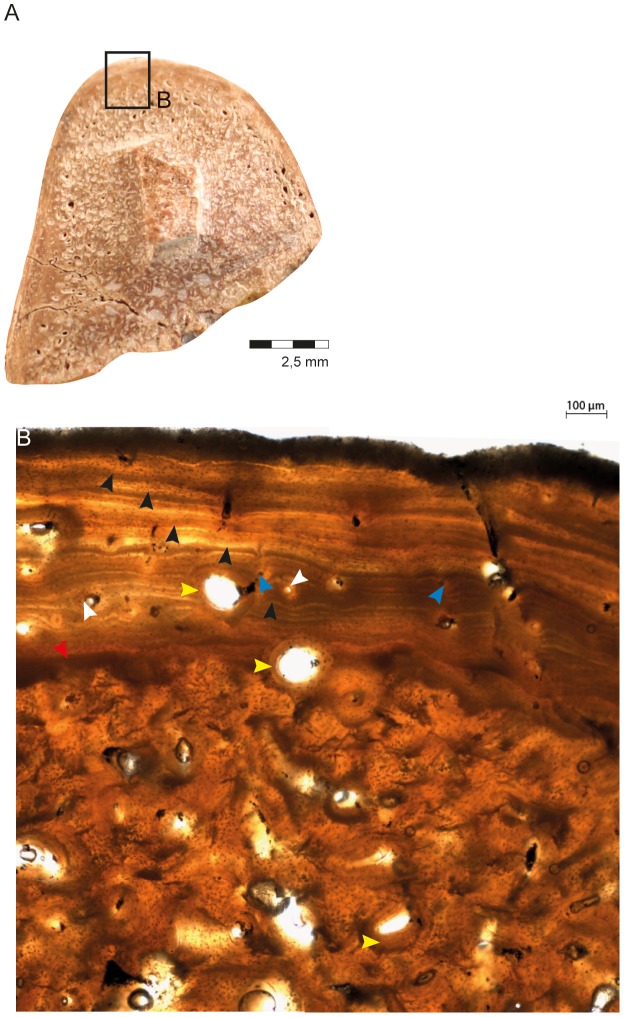
General microstructure anatomy of the tibia CAV 0011-V. (A) View of the complete transect. Black box indicates, respectively, where the related image B was taken. (B) View of the compact periosteal cortex and spongiosa (note the relation of the two tissues). Blue arrows- primary osteons; white arrows- primary osteons; yellow arrows- secondary osteons; black arrows- LAGs and red arrow- transitional area from cortex to remodeled bone.

The tibia preserves only the distal end of the bone, from the metaphysis to the epiphyses (Fig. 2). The diameter of this bone at the point of sampling is 2.94 cm. The sections of this bone are located close to the metaphysis, which is a region of the long bone where periosteal cortices are always thinner than in the midshaft, and the proportion of spongiosa to compact bone is high (Fig. 3A).

The cortex of the tibia, composed of lamellar-zonal bone (LZB), was 1,119.65 µm in width (Fig. 3B). Osteocyte density is consistently high throughout the cortex, especially around the vascular canals. It is higher in the center and inner regions, but decreases somewhat moving periosteally into the avascular regions of the outer cortex. Secondary osteons are observed in the inner and mid cortex, but this is only local remodeling. They increase towards the spongiosa, which shows many resorption rooms. In the spongiosa the secondary osteons occasionally anastomose with each other.

**Figure 4:**
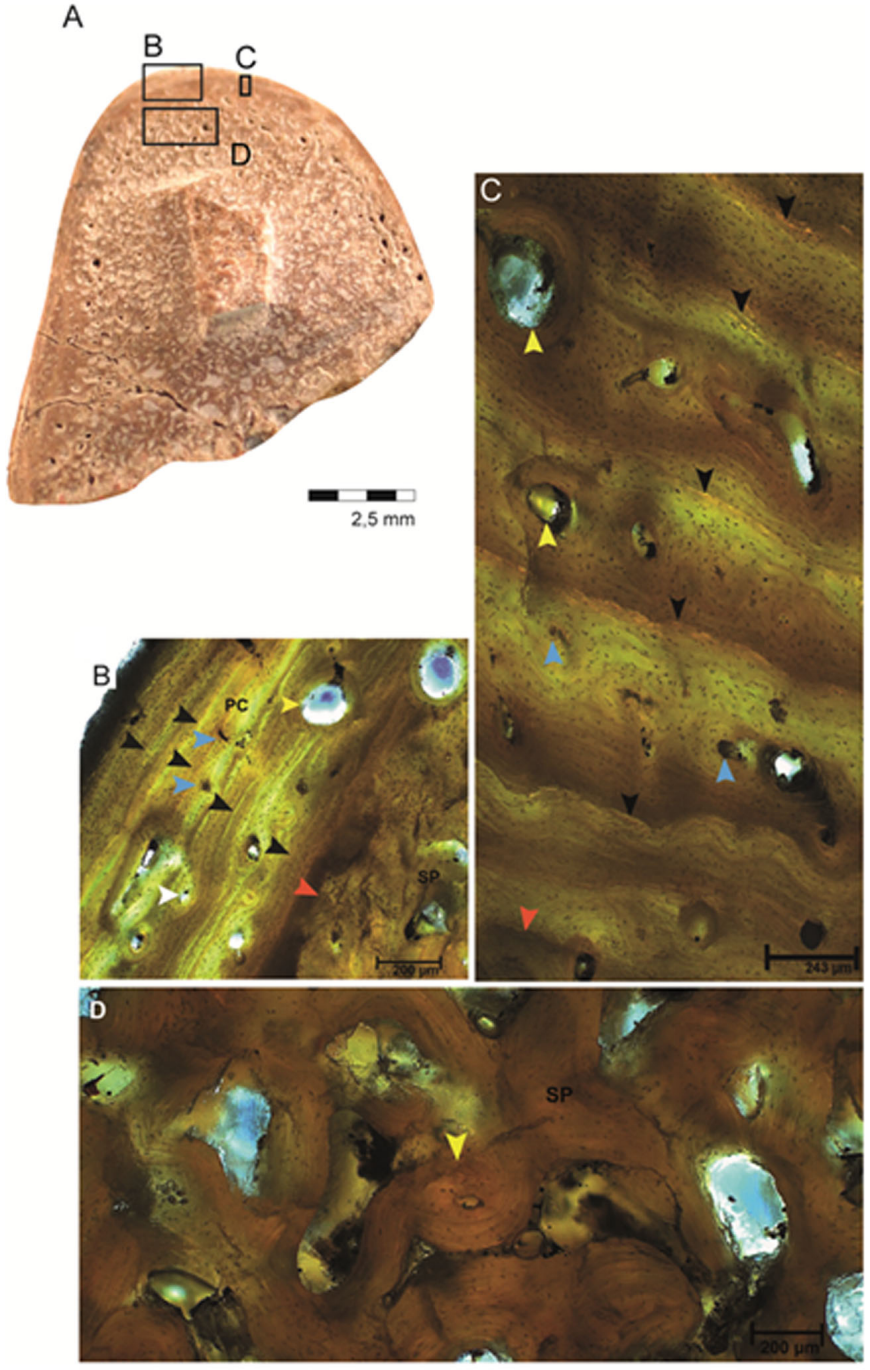
Histological characteristics of the tibia CAV 0011-V. (A) View of the complete transect. Black boxes indicate, respectively, where the related images were taken. (B) View of the cortex exhibiting lamellar-zonal bone tissue with circumferentially organized simple vascular canals (blue arrows), primary osteons (white arrow), secondary osteons (yellow arrow), LAGs (black arrows) and transicional area (red arrow). Periosteal surface to upper left. (C) middle-cortical area showing the five lines of arrested growth (black arrows), secondary osteons (yellow arrows), vascular canals (blue arrows) and contact between the compacta and the spongiosa (red arrow). Periosteal surface to top. The secondary osteons can be found in its inner portion, and the LAGs are indicated by the black arrows. (D) An extended view of the spongiosa where the erosion rooms are visible and infilled by calcite. Rough surfaces of the trabeculae indicate they were formed by resorption. The trabeculae themselves show signs of secondary remodeling, including secondary osteons (yellow arrow). Abbreviations: PC, periosteal compact cortex; SO, secondary osteons; SP, spongiosa.

In the primary bone tissue, the vascular network consists of simple vascular canals and primary osteons that vary in their diameter (from 52.55 µm to 176.77 µm). The orientation of the vascular network is predominantly longitudinal and the canals do not show anastomoses. Five lines of arrested growth are visible (LAGs) (Fig. 4B, C; note black arrows). The zones between them are slightly variable in width around the section, but are generally approximately 300 µm (Fig. 4C). Thus, this region of the bone characterizes 5 cycles (annuli-zones) during the life of the animal. However, sampling at any point except the mid-diaphysis can underestimate the true number of LAGs [32], so it is possible that some cycles may have been obscured by remodeling and resorption.

#### Femur

**Figure 5:**
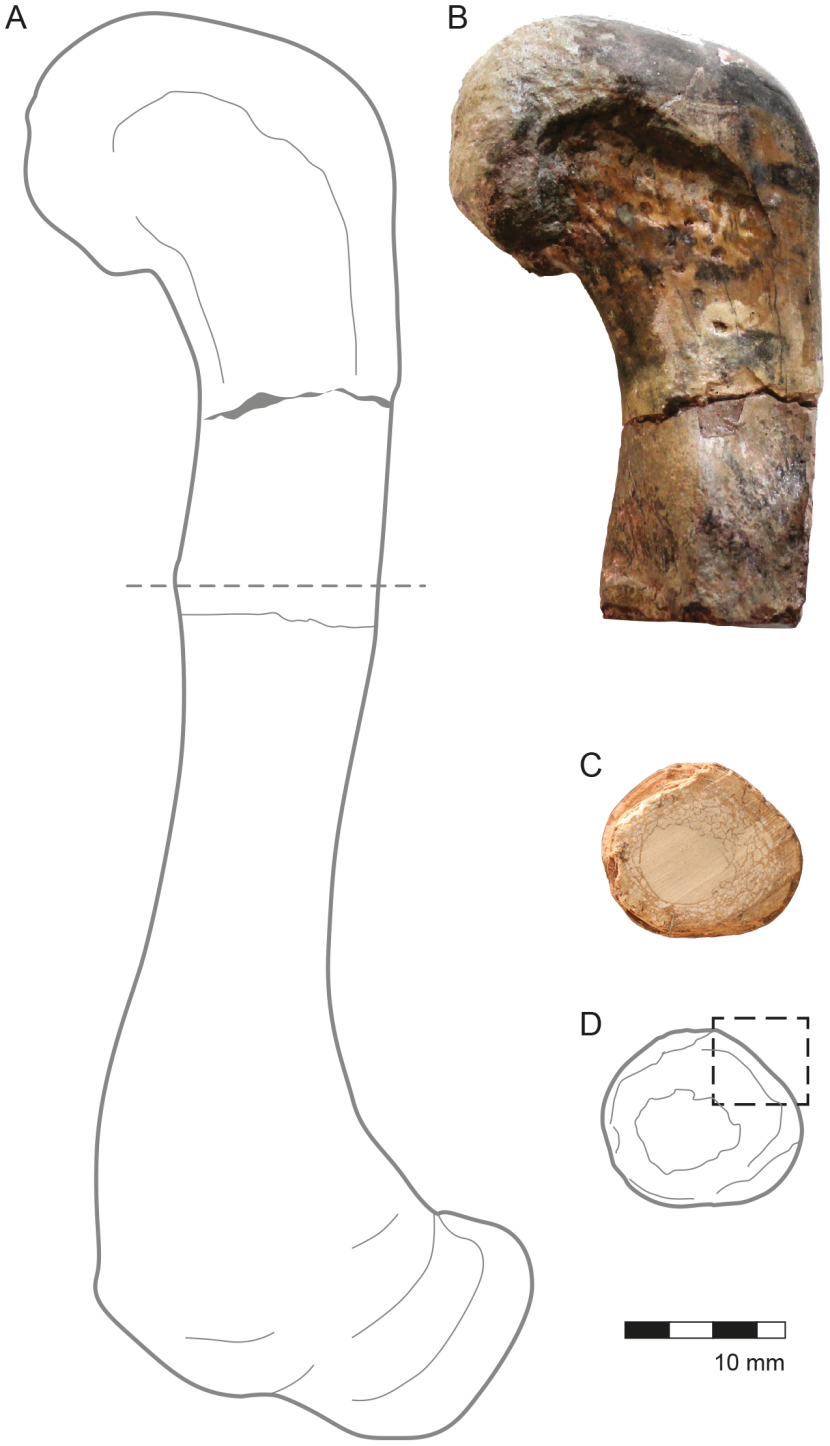
Aspects of the external and internal anatomy in the right femur CAV 0010-V. (A) Drawn reconstruction of the complete femur and the dotted line indicates where the cut was made for the sample collection. (B) Frontal view of the femur proximal portion. (C) A view of the section fabricated for sample collection, which corresponds to a diaphyseal portion where the cortex reaches its region of higher thickness. Note that both the cortex and the spongiosa are of equivalent quantity. (D) The dotted, square, in the draw, represents the region where the analysis and images have been made.

The proximal third of the femur is preserved (Fig. 5B). The diameter of the bone at the point of sampling is 3.10 cm, and the cortex is 1,997.49 µm thick. The sampled section of this bone is located within the diaphysis, closer to the midshaft than the tibia. In this region, the proportion of compact to cancellous bone is always higher than it is in either the metaphysis or the epiphysis (Fig. 5A).

**Figure 6:**
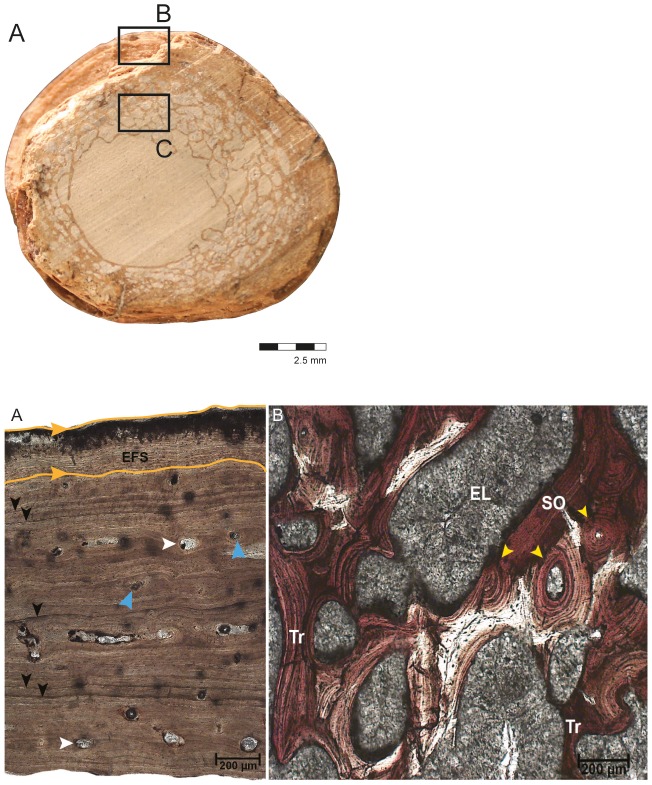
Histological characteristics of the femur, CAV 0010-V. (A) View of the complete transect. Black boxes indicate, respectively, where the related images were taken. (B) A view of the cortex with black arrows indicating one double LAG on the top a single one in the middle and double LAG is also indicating in the bottom and white arrows indicating the primary osteons in this area. The femur exhibits LZB tissue (Lamelar-Zonal Bone) with annuli between the LAGs, along with deposition of the external fundamental system (EFS- delimited by orange lines). (C) Significantly remodeled spongiosa of the femur supported by the presence of secondary osteons and trabeculae. The erosion lacuna is filled with minerals. TR - trabecula; SO - secondary osteons; EL - erosion lacuna.

The femur exhibits a lamellar-zonal histological pattern that is similar to that of the tibia, but has some particularities. Most notably, the femur shows an outermost layer of closely-spaced lines, the external fundamental system (EFS), that characterizes the end of bone growth. The femur also exhibits a different pattern of cortical LAGs in comparison to the tibia. Five lines can be observed, but four of them (two pairs) are double LAGs. One pair is visible in the inner cortex. Moving periosteally, there is a single LAG at midcortex and then another double LAG in the outer cortex. The variation in the zones between LAGs is greater in the femur than in the tibia (Fig. 6B). In the tibia the distance between the LAGs is about 300 µm, whereas in the femur the distance between the closest LAGs are 20 µm and the most distant is 194. In the EFS, there are numerous, closely-spaced LAGs, none of which are very distinct from each other. This prevents an exact number from being determined. As in the tibia, the femur is comprised of LZB, and osteocyte density is consistently high throughout the cortex, especially around the vascular canals. It is higher in the center and inner regions, but decreases moving periosteally into the avascular regions of the outer cortex (Fig. 6B).

The vascular canals vary from 22.12 µm to 85.92 µm in diameter. The orientation of the vascular network is predominantly longitudinal, but unlike the tibia there are some circumferential anastomoses. Secondary osteons are most common in the innermost cortex near the spongiosa, but decrease in density periosteally. The inner and mid cortex show only local remodeling, Secondary osteons diameter ranges, on average, from 150.95 µm to 292 µm. In the spongiosa, the secondary osteons occasionally anastomoses with each other.

## Discussion and Functional Inferences

The morphological specializations of bones in aquatic tetrapods are among the most striking examples of adaptive convergence [33]. Beyond the modification involving the external morphology of their bones, these organisms also share another convergence that is perhaps more striking: the structural specialization exhibited on the inner organization and histological features of their skeletons [34]. These specializations are sufficiently common among the various extant and extinct tetrapod taxa secondarily adapted to an aquatic life that they seem to represent a necessary and unavoidable consequence of this adaptation [35], [36], [17].

The histological pattern (LZB) observed in the femur and tibia consists of primary periosteal deposits composed of bone tissue with simple vascular canals and primary osteons. The presence of cyclic growth marks are conspicuous, partly caused by endogenous physiological rhythms. These rhythms are synchronized and amplified by the seasonal variations in the environment, such as temperature, light, hygrometry, or food availability, indicators of the circannual periodicity of the growth cycles [37], [38], [39]. These indicators are represented by a phase of rapid growth and a subsequent phase of slow growth or temporary growth cessation occurring each year.

The annual periodicity of LAGs has been established empirically in the long bones of living crocodylians. Osteoderms of Nile crocodiles have been shown to be deposited in annual cycles, as well. Juveniles injected with tetracycline were shown to deposit zones during the hot season, whereas annuli corresponded to the cool seasons. Therefore, the presence of one zone and one annulus marks the passage of one year in Nile crocodiles [39]. Annual LAGs are common in many living and extinct crocodyliforms, for example, in the limbs of the marine Thalattosuchia [17]. Therefore, we infer these cycles are annual in the dyrosaurid elements examined here.

The femur preserves three annual cycles: the two endosteal and periosteal double-LAGs, and a midcortical single LAG. Double LAGs, which appear as two very closely adjacent twin lines. When this pattern is in the sample, the question of two growth cycles arises; however, these twin LAGs may be counted as a single one representing one year [40]. In the EFS, LAGs can be observed but are closely-spaced and difficult to count. The presence of an EFS indicates that this animal was more or less fully grown at time of death, having completed the active growth phase. We cannot definitively conclude that this is the real number of LAGs, because the midshaft was not preserved, but it has been sampled very close. These data provide more complete information when compared to the tibia which was sampled further from the mid-diaphysis. The tibia showed 5 LAGs and no evidence of double-LAGs, indicating that these cycles represent five distinct moments of slow growth (annuli-zone). These analyses were, similarly, used by Woodward et al., (2013) [41] when they sampled American alligator and found evidence of slow growth and EFS in these captive animals. EFS is a rare structure in crocodilians but it has been found in several Triassic pseudosuchians [41]. The EFS, here reported for the first time in the Dyrosauridae, support the hypothesis that determinate growth may be the rule rather than the exception for sauropsids [41].

It is assumed in this report that these crocodyliforms were not juveniles. Based on the extent of the trabecular bone and the limited number of secondary osteons in the cortex, we conclude that the tibia (CAV 0010-V) belonged to a young adult. The femur (CAV 0011-V) was likely a senescent adult based on the observations of the spongiosa/remodeling processes and the presence of secondary osteons in the cortex. The deposition of EFS layer, in the femur, is also a signal of the end of active bone growth and the onset of skeletal senescence [42]. This indicates that this animal had reached its upper growth asymptote at time of the death [42]. The absence of an EFS in the tibia suggests that the upper growth asymptote had not been reached and the specimen is still in active growth [43]. This is supported by the fact that the LAGs are preserved around the section, and that these animals did not have their cortex significantly remodeled compared to a typical cortex from an older individual, for example in the femur. The blood supply, when present, is arranged in the concentric layers of the longitudinal canals with a certain rate of remodeling that is thought to be responsible for the recycling of fixed calcium in response to the general physiologic needs of the organism. This recycling of calcium is revealed by the presence of secondary osteons and erosion lacunae. This basic type of tissue is by no means exclusive to the Thalattosuchia and Dyrosaridae; such tissue is also encountered in numerous extant and extinct poikilothermic tetrapods, including stegocephalians [44], [45] and chelonians [46], [47].

In *Dyrosaurus phospaticus*, the histological structure of the cortex is also characterized by the presence of LAGs [18]. The structure suggests a cyclic pattern of growth similar to the specimens described above. However, the spongiosa of the vertebral center in *Dyrosaurus phospaticus*, compared to an extant taxon, (e.g. *Crocodylus niloticus)*, exhibits more cavities [18]. Additionally, the bone trabeculae of *D. phospaticus* are thinner compared to *C. niloticus*. Limb bones experience different growth and biomechanical patterns compared to vertebrae and skull bones. In limb bones, such observations and comparisons also characterize an adaptation to an aquatic life. Although this lifestyle has been proposed for dyrosaurs, these are the only specimens sampled for histological studies to date [18].

The histological and physiological analysis of dyrosaurids in this study is similar to those of another group of marine crocodiles, the Thalattosuchia. In this clade, the histology is largely similar to that of CAV 0010-V and of *Dyrosaurus phospaticus* [18]. Two types of structural organization of bone occur in aquatic tetrapods: osteoporotic [48] or pachyostotic. Osteoporotic bone has a very porous/spongy inner structure and is characterized by a loss of bone through accelerated resorption rates. An animal with osteoporotic bones would be better suited for faster swimming [48] because the reduction of bone tissue increases the animal’s maneuverability in water. This pattern is exemplified by extant cetaceans and marine turtles, which swim swiftly [48], and also occurs in ichthyosaurs [48]–[50].

Pachyostotic bone involves a local or general increase in skeletal mass and can be caused by three mechanisms: osteosclerosis (inner compaction of bone), pachyostosis (hyperplasy of compact cortices), or a third mechanism that combines the two previous ones, known as pachyosteosclerosis [17]. [51]. The resulting increase in skeletal mass is considered to play the functional role of ballast for buoyancy control and hydrostatic regulation of body trim [51], [52].

According to Houssaye (2009) [52], true pachyostosis (cortical hyperplasy) is observed in tetrapods secondarily adapted to life in water and those that live in shallow marine environments [51], [52]. Osteosclerosis is observed in fully aquatic forms only. Coastal forms (organisms that are poorly adapted to rapid, sustained swimming for anatomical and/or physiological reasons), exhibit both osteosclerotic and pachyostotic tissue [53]. Truly pachyostostic taxa include Tangasaurus and Hovasaurus [52], [54], pachypleurosaurs [52], nothosaurs [52], [55], and Ophidiomorpha [52], [56]. Taxa that increase bone mass by osteosclerosis include *Claudiosaurus*, some derived mosasauroids and *Placodus* [52].

Notably, none of the marine or aquatic crocodylomorphs previously examined (Crocodylus, Thalattosuchians, Pholidosauridae and Dyrosauridae seem to display true pachyostosis [52], although osteosclerosis is common in Thalattosuchia [17].

The tibial metaphysis (CAV 0010-V) has very thin compact cortical bone and extensive trabeculae filling most of the bone in cross-section. Although this pattern is observed in osteoporotic bone, it is also typical of long bone metaphyses, so the ecology cannot be inferred from this section (Fig. 4A).

In the proximal diaphysis of the femur (CAV 0011-V), the cortical compacta is much thinner compared to mid-diaphyseal sections of extant crocodylians [41]. Although this region is naturally expected to display a slightly thinner cortex, it is surprisingly thin despite being sampled much closer the midshaft. Additionally, the trabeculae here are extensive and fill most of the medullary cavity, despite being close to the mid-diaphysis. These trabeculae extend throughout the area normally filled by compact bone, leave a clear medullary cavity (Fig. 6A), and were clearly formed by the resorption and remodeling of primary tissues (Figure 6C). This is consistent with osteoporosis, suggesting a fast-swimming ecology for this animal. There is also some lamellar thickening of the trabeculae in the femur, which may result from the normal “finishing off” of trabeculae during remodeling, but is also consistent with osteosclerosis. If this is the case, osteosclerosis is minimal in these dyrosaurids. However, as osteosclerosis is associated with a fully aquatic lifestyle, this is not incompatible with a fast-swimming ecology.

The histological, ecological, and physiological inferences of the dyrosaurids examined in this study are similar to those of another group of marine crocodiles, the Thalattosuchia and to descriptions of other skeletal elements of *Dyrosaurus phospaticus* [18]. Among the thalattosuchians, observations of the femoral microstructure are especially similar to that described for the Teleosauridae, a group that is clearly aquatic based on morphology and histology, but were not as well adapted as the obligatorily marine animals, such as mosasaurs, elasmosaurs, and metriorhynchid thalattosuchians [33], [17]. In general, dyrosaurids have been reconstructed as being shallow, near-shore marine animals utilizing axial swimming typical of extant crocodylians, with perhaps great tail undulatory frequency and more powerful forward thrust generated by expanded muscles of the tail [57]. These hypotheses are consistent with the histological pattern found here. Therefore, we suggest an aquatic marine condition for the dyrosaurids from the Paraíba Basin, but not one with specialized adaptation for fully marine life.

Although these results are consistent with the histology in anatomically convergent taxa, it will be necessary to make additional sections from the mid-diaphysis in order to confirm that these animals exhibit osteoporosis or osteosclerosis and assign their ecology with more confidence [41], [48].

## Conclusion

The marine habit was already proposed to the Dyrosauridae. It was based mainly in evidences of body modifications, and for the marine paleoenvironment deposits where the remains are found. The microstructural composition with patterns consistent with osteoporosis can enforce that these animals correspond to animals with adaptations tending to a semi-aquatic life and fast swimming.

This is the first information so far of long bone histological analyses in the Dyrosauridae, and with Thalattosuchia constitutes the only paleohistological evidences about the basal neosuchians. Further analysis, specially using the midshaft information, and from others representatives from this family can strengthen these evidences.
